# Supplementary material for: α-Helical peptidic scaffolds to target α-synuclein toxic species with nanomolar affinity
Source: Nat Commun. 2021 Jun 18;12:3752. doi: 10.1038/s41467-021-24039-2 (PMC8213730; doi:10.1038/s41467-021-24039-2)
Supplement: Supplementary file 1 — Supplementary Information [file 41467_2021_24039_MOESM1_ESM.pdf]

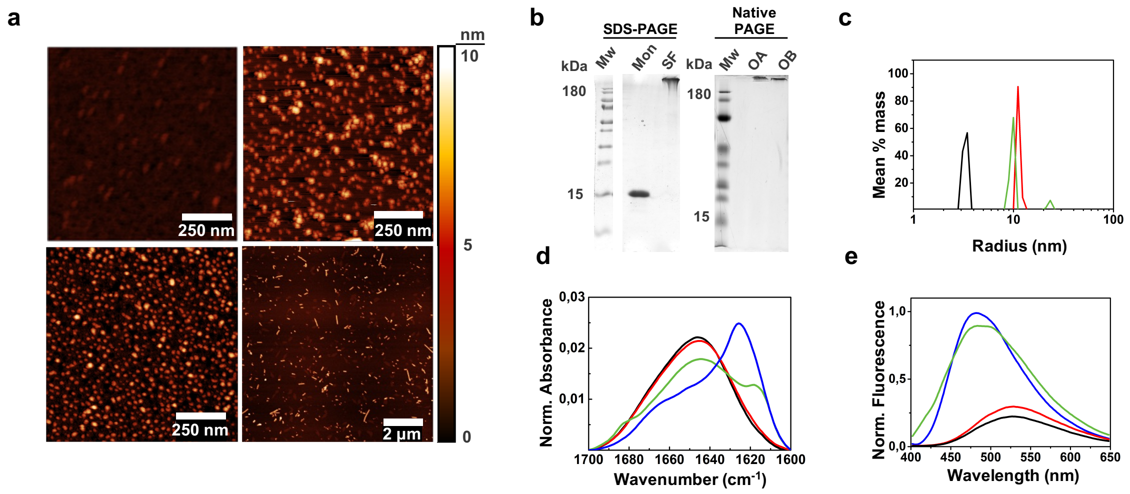

Supplementary Figure 1. **Characterization of α–synuclein species.** The size, morphology, purity, structure and hydrophobicity of the α–synuclein (αS) species used in this study were analyzed in order to provide a molecular basis for further result interpretation. (**a**) AFM analysis of monomeric αS (top left), type A* (top right) and type B* (bottom left) oligomers and sonicated fibrils (bottom right) are shown. Statistical size distribution analysis yielded a 5.1 ± 0.4 nm height and 28 ± 6 nm diameter for type A* oligomers, 4.4 ± 0.9 nm height and 32 ± 5 nm diameter for type B* oligomers, and a 6.3 ± 0.3 nm height, 95 ± 14 nm width and 300 ± 140 nm length for sonicated fibrils. Scale bar and height color code are shown. Results are consistent between two independent replicates. These data are in good agreement with the diffusive behavior of the αS species as mentioned in the article and also in agreement with previously published data[1]. (**b**) Electrophoretic behavior and purity of the αS species in denaturing (left) or native PAGE (right). No fragments or other contamination are visible in the sample. Importantly, no monomeric αS (referred to as Mon in the figure) is visible in the oligomeric (in both type A* and type B* oligomers, referred to as OA and OB, respectively, in the figure) or fibrillar protein preparations (referred to as SF in the figure). Results are consistent between two independent replicates. Aggregated species are larger than 180 kDa and thus do not enter the wells, in agreement with previously published data[1]. (**c**) DLS analysis of αS species. A fairly homogeneous size distribution is visible for monomeric αS (black) as well as type A* (red) and type B* (green) oligomers. The fibrillar αS samples showed a very large polydispersity index preventing their analysis by this technique. Size distributions are given in % mass. (**d**) Normalized infrared (IR) spectra of monomeric αS (black), type A* oligomers (red), type B* oligomers (green) and fibrils (blue). A clearly disordered conformation can be seen for the monomer and type A* oligomers while a substantial β-sheet structure is observed in the type B* oligomers, if less than in the fibrils, as expected and reported previously[1,2]. In particular, deconvolution analysis yielded a β-sheet

content of 0 % for monomer and type A* oligomers, 35 % for type B* and 56.4 % for fibrils, also in good agreement with previously published data[1,2]. (**e**) ANS binding analysis of αS species. The normalized fluorescent spectra of the different samples show a higher hydrophobicity, seen as an enhanced fluorescence emission and a spectral blue-shift of type B* oligomers (green) and fibrils (blue) compared with monomeric (black) αS and type A* oligomers (red), which show a similarly low intensity and are not blue-shifted. This is in good agreement with previously published data[1,2]. Unprocessed scans of the gels are presented in the Source Data file.

a

Residue	Wt	1	2	3	4	5	6	7	8	9	10	11	12	13	14	15	16	17	18	19	20	21	22
α-helical predicted content	2.6	3.2	1.8	1.8	1.9	1.1	0.9	0.4	0.6	0.4	0.4	0.7	0.7	0.6	0.6	0.5	0.8	1.4	2.0	2.9	2.3	2.5	2.9

b

PSM*α*3 MEFVAKLFKFFKDLLGKFLGNN
dPSM*α*3 MEFVAKLFPFPKDLLGKFLGNN
* *

c **d**

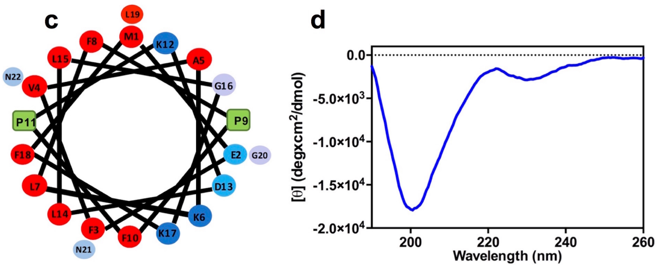

Supplementary Figure 2. **Design of a non-amphipathic PSMα3 variant (dPSMα3).** (**a**) Computational proline scanning. Predicted α-helical propensity according to the AGADIR score; higher values indicate higher predicted α-helical propensity. (**b**) Sequence alignment of PSMα3 and dPSMα3. (**c**) Helical wheel projection of dPSMα3 sequences showing the theorical location of the introduced prolines (green) (red, hydrophobic residues; blue pallet, hydrophilic residues depending on their character). (**d**) Far-UV circular dichroism spectrum of dPSMα3.

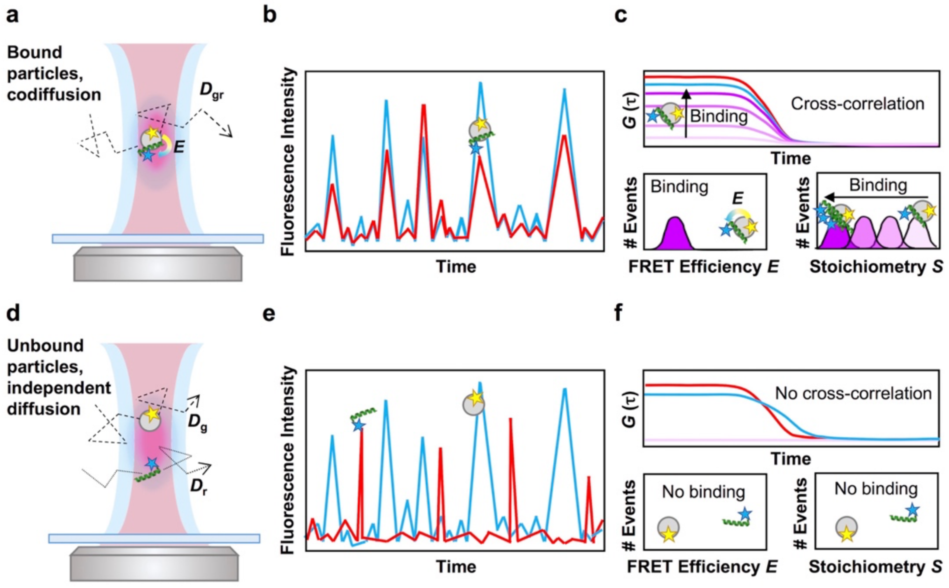

Supplementary Figure 3. **Schematic figure showing the dual-color time-resolved fluorescence spectroscopy approach to characterize the binding of the peptides to the different α-synuclein species**. The upper panels (**a-c**) illustrate a scenario where binding occurs whereas the lower panels (**d-f**) serve as an example of a non-binding scenario. (**a**) Two interacting molecules labeled with a green and a red dye (depicted as a yellow or blue star, respectively) freely co-diffuse through the dual-laser confocal volume. The co-diffusion of the molecules is indicated as D_{gr} while FRET between the dyes in the complex is indicated as *E*. (**b**) Illustration of a fluorescence time-trace of the co-diffusing molecules where intensity bursts of the green and red detection channels (blue and red traces, respectively) coincide in time. (**c**) the upper panel illustrates a positive cross-correlation scenario (purple lines) where the cross-correlation amplitude (*G*) is directly proportional to the degree of binding. The bottom left panel depicts a FRET efficiency (*E*) distribution from interacting molecules while the bottom right panel shows the green dye-to-red dye (donor-to-acceptor) fluorescence stoichiometry of those interacting particles and shows how the stoichiometry decreases with an increasing binding degree. (**d-f**) illustrate the same parameters in the case where no interaction is observed.

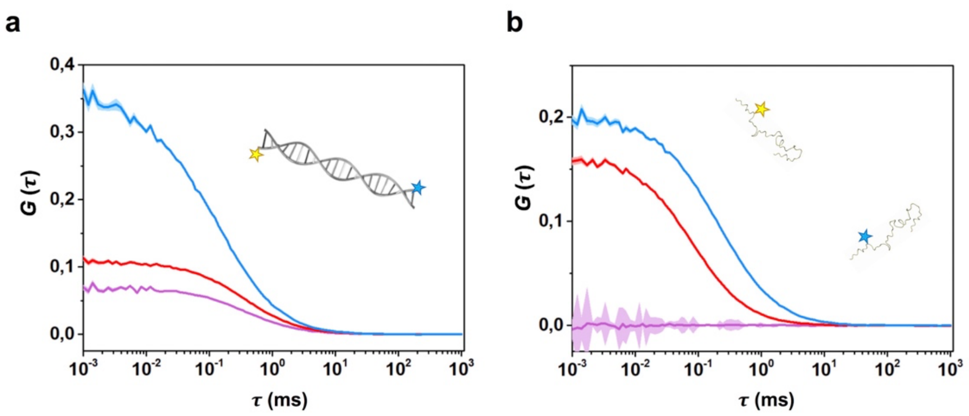

Supplementary Figure 4. **Fluorescence cross-correlation spectroscopy positive and negative control.** Auto-correlation curves of AF488 (blue) and Atto647N (red) and cross-correlation curves (purple) of samples containing (**a**) 10 nM of doubly-labelled dsDNA molecule or (**b**) 15 nM of non-interacting AF488-αS and Atto647N-αS (15 nM each). The amplitude (*G*) error is shown as faint colored area for the corresponding correlation curves.

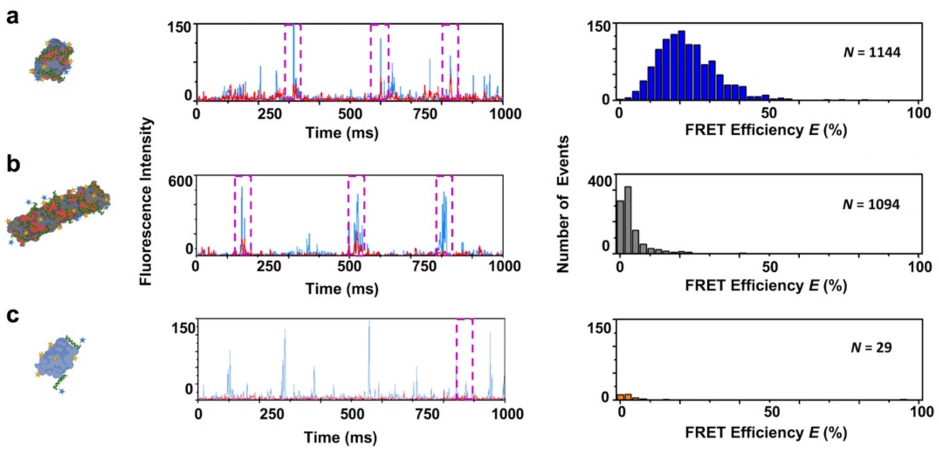

Supplementary Figure 5. **α-Synuclein-PSMα3 binding analyzed by fluorescent single-particle spectroscopy.** Representative intensity time traces (left panels) and intensity-calculated FRET efficiency histograms (right panels) for samples containing (**a**) ~1 nM αS type B* oligomers and ~5 nM PSMα3, (**b**) ~5 nM αS fibrils and ~5 nM PSMα3 and (**c**) 1 nM αS type A* oligomers and ~5 nM PSMα3 (concentrations expressed as protein/peptide mass concentrations; particle concentrations in the range of pM). In the intensity traces, events displaying both donor and acceptor intensities above *α*S monomer threshold (see materials and methods) are shown in purple dashed boxes. These events were then used to calculate the intensity-based FRET efficiency *E* histograms. The total number of FRET events, *N*, used to calculate each histogram is shown in each panel. These results show, directly from the intensity raw data, the high avidity of both PSMα3 for either type B* oligomers or fibrils (**a-b**) and the low ability to bind non-toxic aggregated species like the type A* oligomers (**c**). Acquisition times were the same for all data shown.

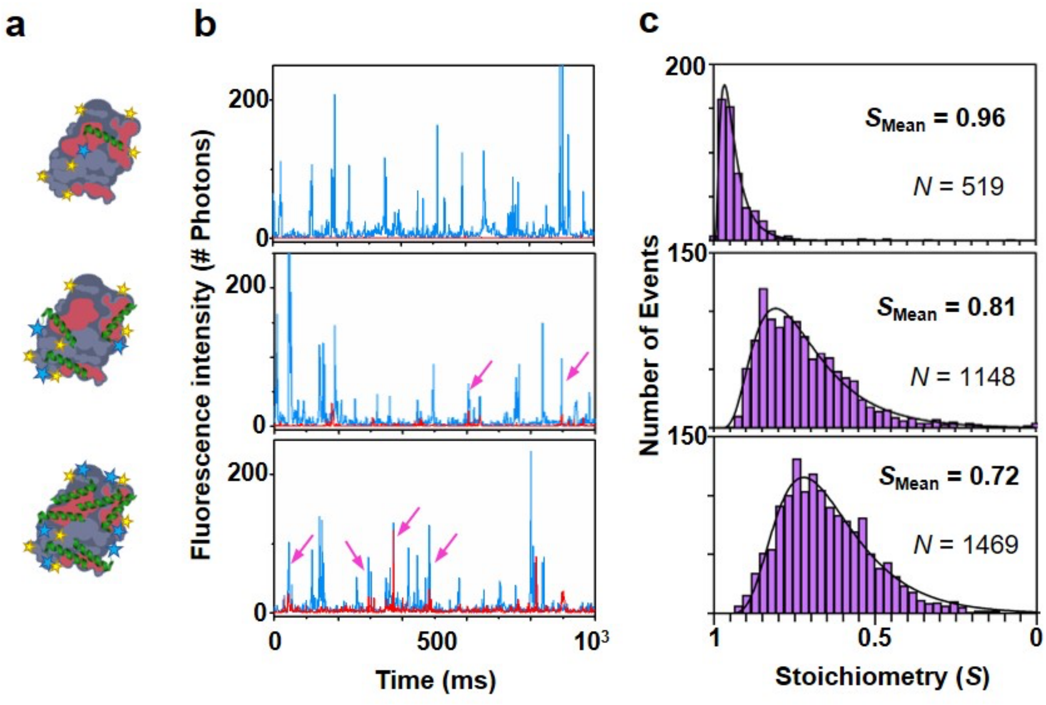

Supplementary Figure 6. **Single-particle fluorescence stoichiometry analysis.** Data is provided to illustrate the experimental approach leading to a burst-wise fluorescence stoichiometry analysis which complements the dcFCCS analysis to obtain the binding curves shown in this work. **a)** Schematic representations of type B* oligomers bound to increasing (top to bottom) concentrations of PSMα3. **b)** Intensity-based raw data corresponding to 1-second time frames (intensity time traces) of binding experiments with ~1 nM type B* oligomers (blue) and ~0.1 nM (top), ~2 nM (middle) and ~10 nM (bottom) PSMα3 (red). Note that the concentrations are given as protein/peptide mass concentrations and that particle concentrations are significantly lower, always under single-particle regime (for example in the case of type B* oligomers, 1 nM of mass concentration corresponds to ~30 pM oligomer particle concentration). Two-color coincident events that were intensity threshold-selected for stoichiometry analysis are shown with pink arrows in the figure panels. **c)** Fluorescence stoichiometry distributions from the experiments shown in **b)**. Increasing PSMα3 concentrations yield lower stoichiometry values as more peptide molecules can be bound to one oligomer. The log normal-fitted mean stoichiometry value (S_{mean}) is shown. The total number of events (*N*), which increases with increasing PSMα3 concentrations, is also shown. Acquisition times were the same for all data shown.

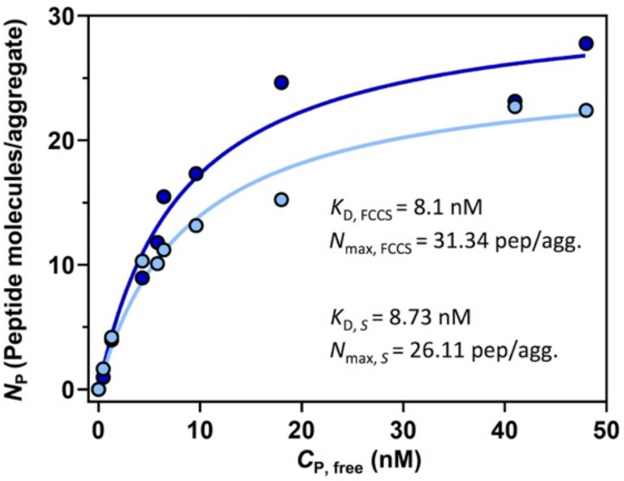

Supplementary Figure 7. **Comparison of the titration binding curves of α–synuclein type B* oligomers with PSMα3 peptide obtained by dcFCCS or dual-color single-particle fluorescence spectroscopy analysis.** The number of peptide molecules bound to one oligomer (N_P) at increasing peptide concentrations was calculated independently by dcFCCS (dark blue circles) or fluorescence stoichiometry analysis in dual-color single-particle fluorescence experiments (light blue circles), yielding very similar titration binding curves that resulted in very similar binding parameters when analyzed using a model of *n* identical and independent binding sites per α-synuclein (αS) aggregated species (solid lines). The fitted parameters K_D and N_{max} are also shown for each analytical approach. These results show how two different analytical methods, one which correlates fluorescence fluctuations over whole time traces and another one which analyzes single fluorescent bursts, can be applied to obtain very similar binding parameters, thus validating our strategy.

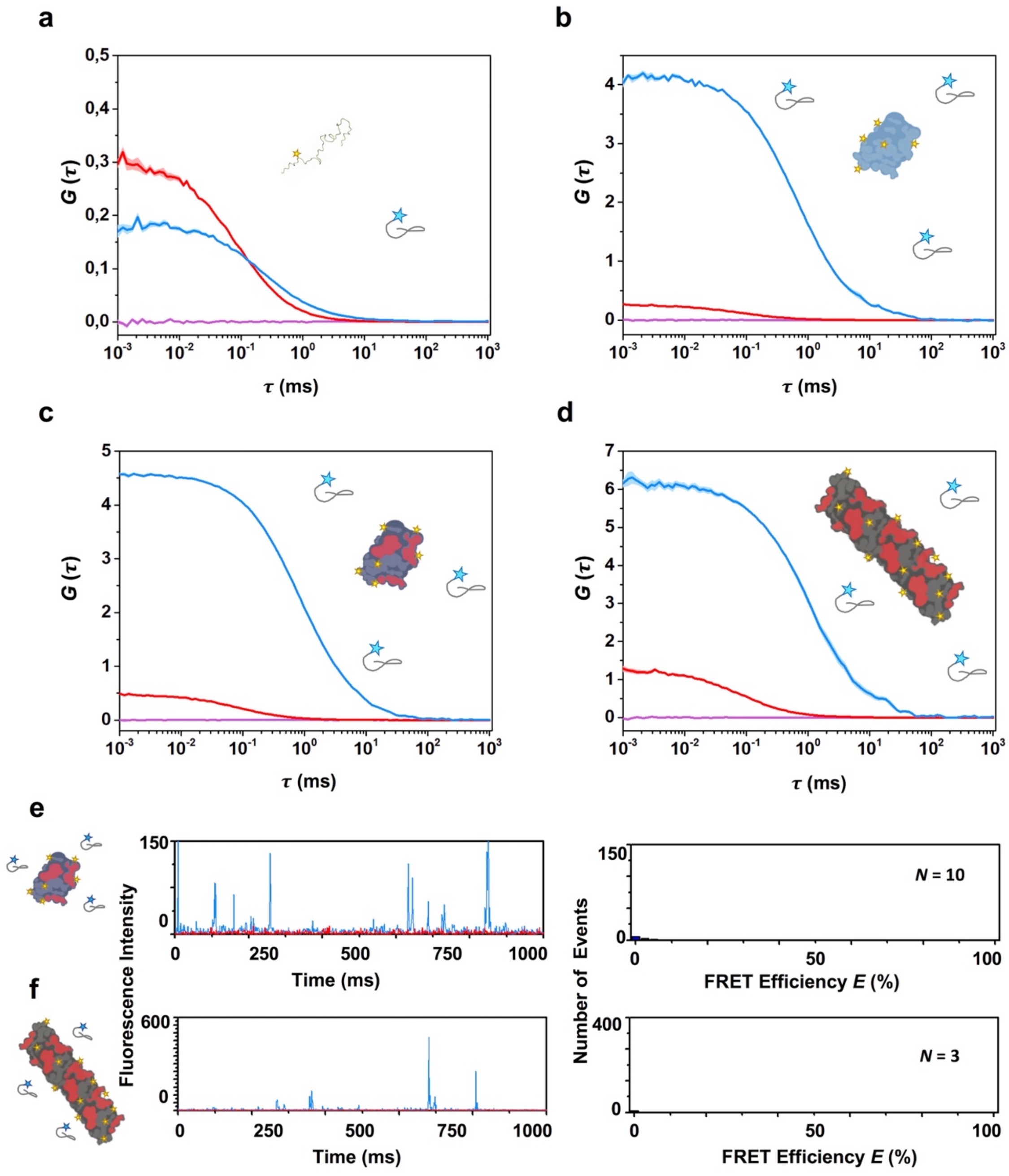

Supplementary Figure 8. **Interaction of dPSMα3 with the different αS species.** (**a-d**) Auto-correlation curves for α–synuclein (αS) and dPSMα3 and cross-correlation curves for interacting molecules are shown in blue, red and purple lines, respectively. The amplitude (*G*) error is shown in faint blue, red and purple, respectively. ~15 nM αS monomer (**a**), ~1 nM type A* (**b**), type B* (**c**) oligomers and sonicated fibrils (**d**) were allowed to interact with ~15 nM dPSMα3. No cross-correlation is observed in any case. (**e-f**) αS-dPSMα3 binding analyzed by dual-color single-particle fluorescent spectroscopy. Representative intensity time traces (left panels) and intensity-calculated FRET efficiency histograms (right panels) for samples containing (**e**) ~1 nM αS type B*

oligomers and ~5 nM dPSMα3, (**f**) ~5 nM αS fibrils and ~5 nM dPSMα3. FRET single-particle fluorescence data directly show the inability of dPSMα3 to interact with either type B* oligomers or fibrils (**e, f**), as just few, if any, events were observed in comparison to the experiments with the PSMα3 peptide. Acquisition times were the same for all data shown.

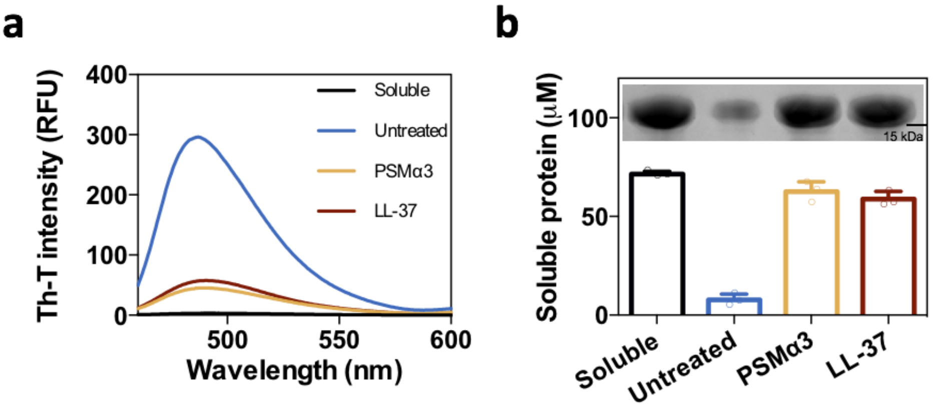

Supplementary Figure 9. **Orthogonal validation of PSMα3 and LL-37 anti-aggregational activity.** (**a**) Thioflavin-T fluorescence spectra in the presence of monomeric α–synuclein (αS) (Soluble) and end-point αS aggregation reactions (70μM) performed in the absence (Untreated) and in the presence of 35 μM of PSMα3 or LL-37. Spectra were recorded from 460 to 600 nm with an excitation wavelength of 445 nm. (**b**) Characterization of the amount of soluble αS in end-point aggregation reaction samples after sedimentation. The same samples were analyzed by SDS-PAGE (top panel) and quantification was performed by measuring the absorbance at 280 nm (ε = 5960 M^{-1} cm^{-1}). Protein quantities were measured in triplicate. Data were expressed as mean ± S.D (n = 3 independent experiments). The black line indicates the position of the 15 kDa band of the protein ladder. Unprocessed scans of the gels are presented in the Source Data file.

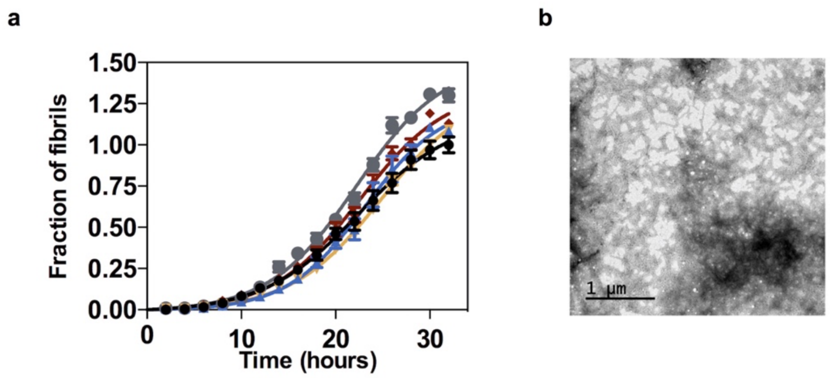

Supplementary Figure 10. **Effect of dPSMα3 on *in vitro* α–synuclein amyloid fibrillation.** (**a**) Aggregation kinetics of 70 μM α-synuclein (αS) and titration of the inhibitory activity of dPSMα3 at different concentrations: 35 μM (green), 14 μM (orange), 7 μM (blue), 3.5 μM (gray) and in the absence of dPSMα3 (black). Data were expressed as mean ± s.e.m (n = 9 independent experiments). (**b**) TEM micrograph of the end point of the aggregation kinetics in the presence of 70 μM of dPSMα3.

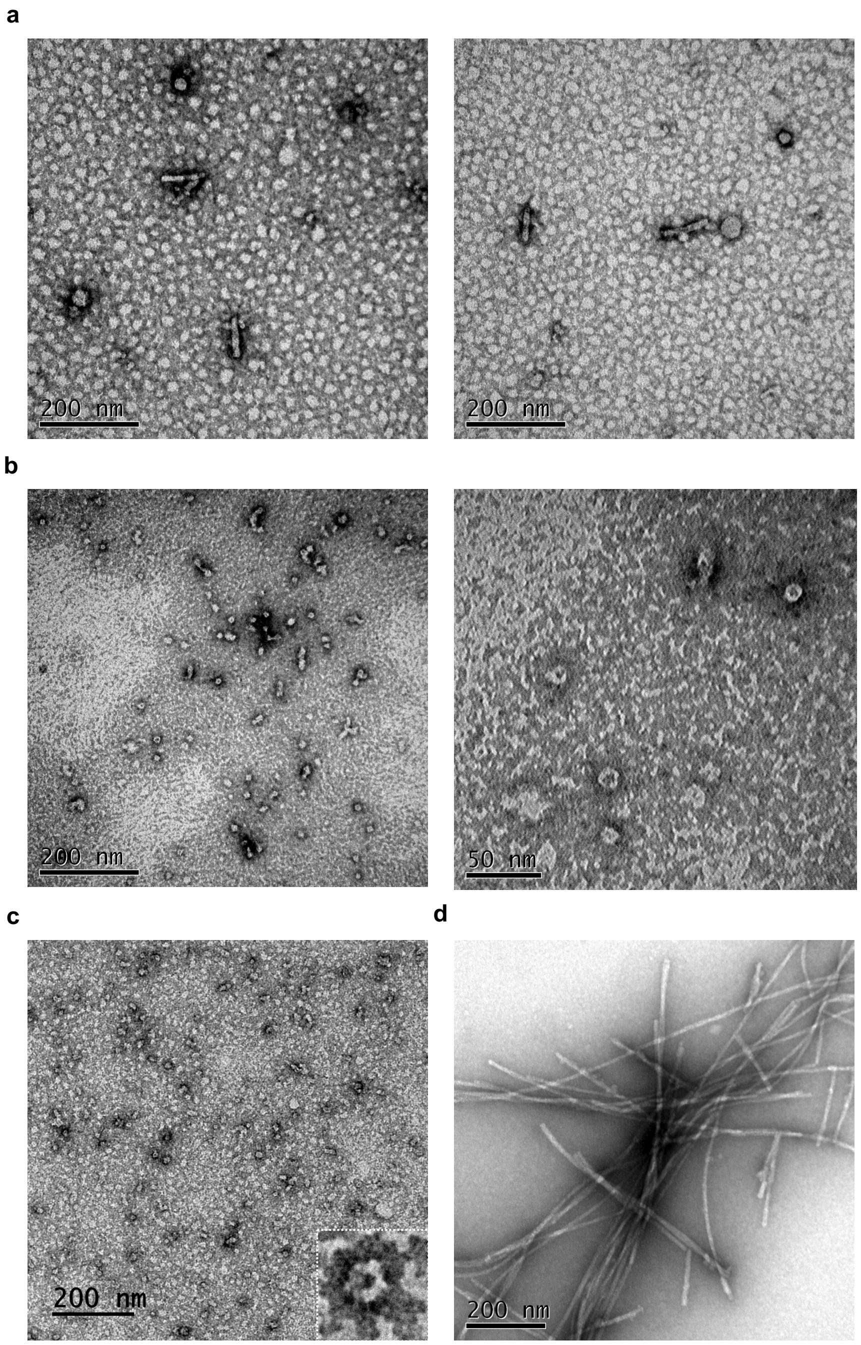

Supplementary Figure 11. **Representative TEM micrographs of α-synuclein low molecular weight aggregates.** Low molecular weight aggregates of α-synuclein (αS) aggregated for 12 hours in the absence (**a**) and the presence (**b**) of PSMα3. (**c**) Type B* oligomer preparation. Inset shows a type B* oligomer at high magnification. (**d**) End point αS amyloid fibrils. Results are consistent between two independent replicates.

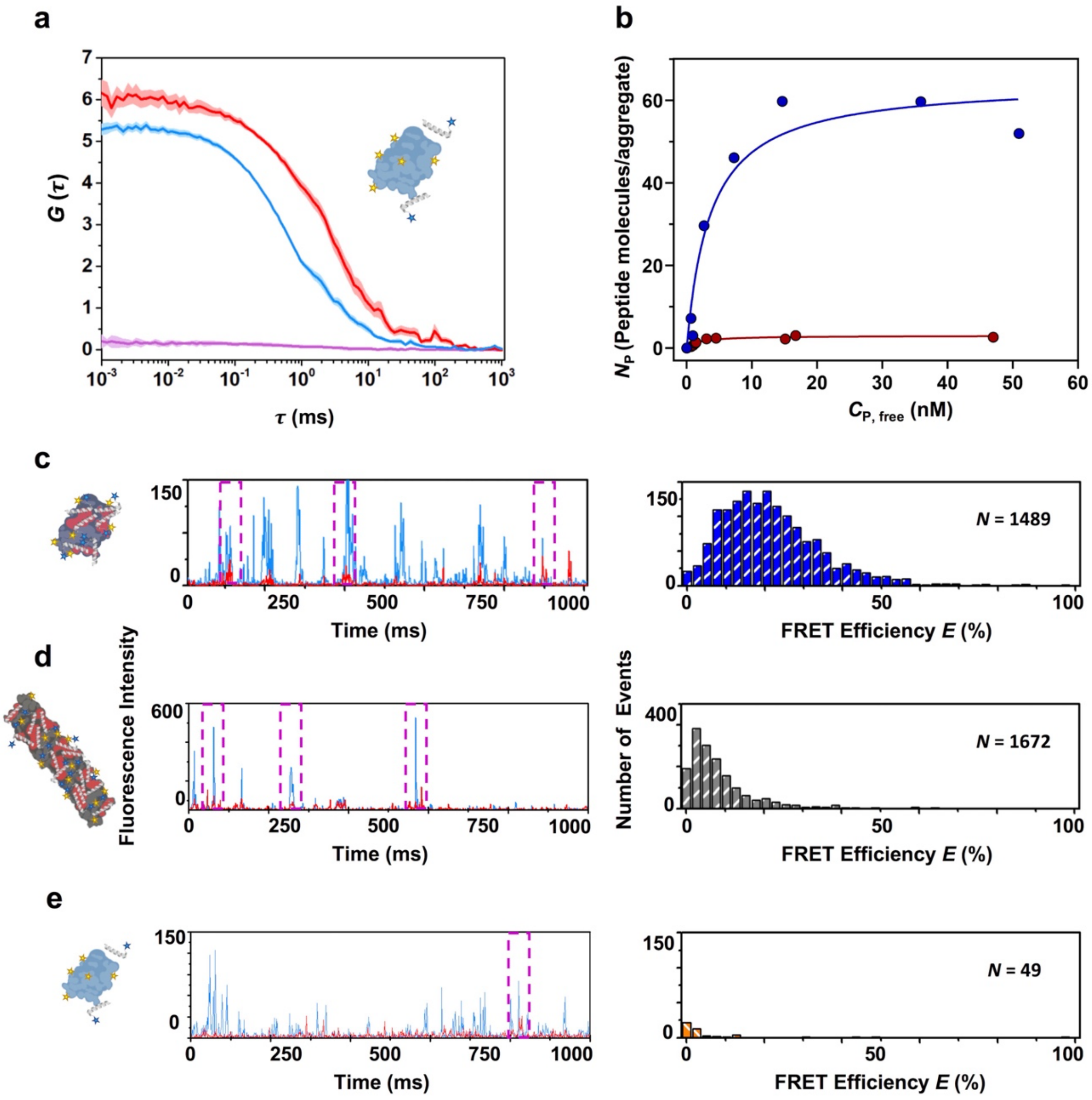

Supplementary Figure 12. **Interaction of LL-37 with α–synuclein aggregates by FCCS and fluorescent single-particle spectroscopy.** (**a**) Auto-correlation curves for α-synuclein (αS) (blue) and LL-37 (red) and cross-correlation curve for the interacting molecules (purple) in samples containing ~1 nM type A* oligomers and ~5 nM LL-37 peptide. The amplitude (*G*) error is shown as faint colored area for the corresponding correlation curves. (**b**) Titration binding curves for the interaction of LL-37 with type A* oligomers (red circles) or type B* oligomers (blue circles) obtained by dcFCCS, showing their corresponding analysis assuming a model of *n* independent binding sites per *α*S aggregated species (solid lines). (**c-e**) αS-LL-37 binding analyzed by Fluorescent single-particle spectroscopy. Representative intensity time traces (left panels) and intensity-calculated FRET efficiency histograms (right panels) for samples containing (**c**) ~1 nM αS type B* oligomers and ~5 nM LL-37, (**d**) ~5 nM αS fibrils and ~5 nM LL-37 and (**e**) ~1 nM αS type A* oligomers and ~5 nM LL-37. Acquisition times were the same for all data shown.

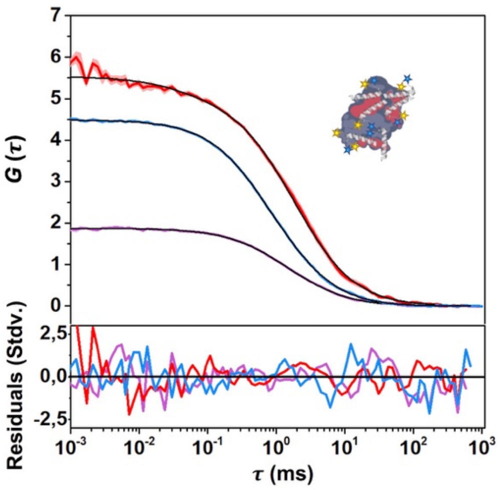

Supplementary Figure 13. **Fitting of fluorescence correlation and cross-correlation data.** Representative auto-correlation and cross-correlation curves of a sample of 1 nM type B* oligomers and 5 nM LL-37 peptide are shown in blue, red and purple lines, respectively. The amplitude (*G*) error is shown as faint colored area for the corresponding correlation curves. Best fits to 1-diffusion component (cross-correlation) or 2-diffusion component (auto-correlations) simple diffusion models are shown as black lines. The residual analysis of the best fits is also shown as standard deviation in colored lines for each correlation curve fit.

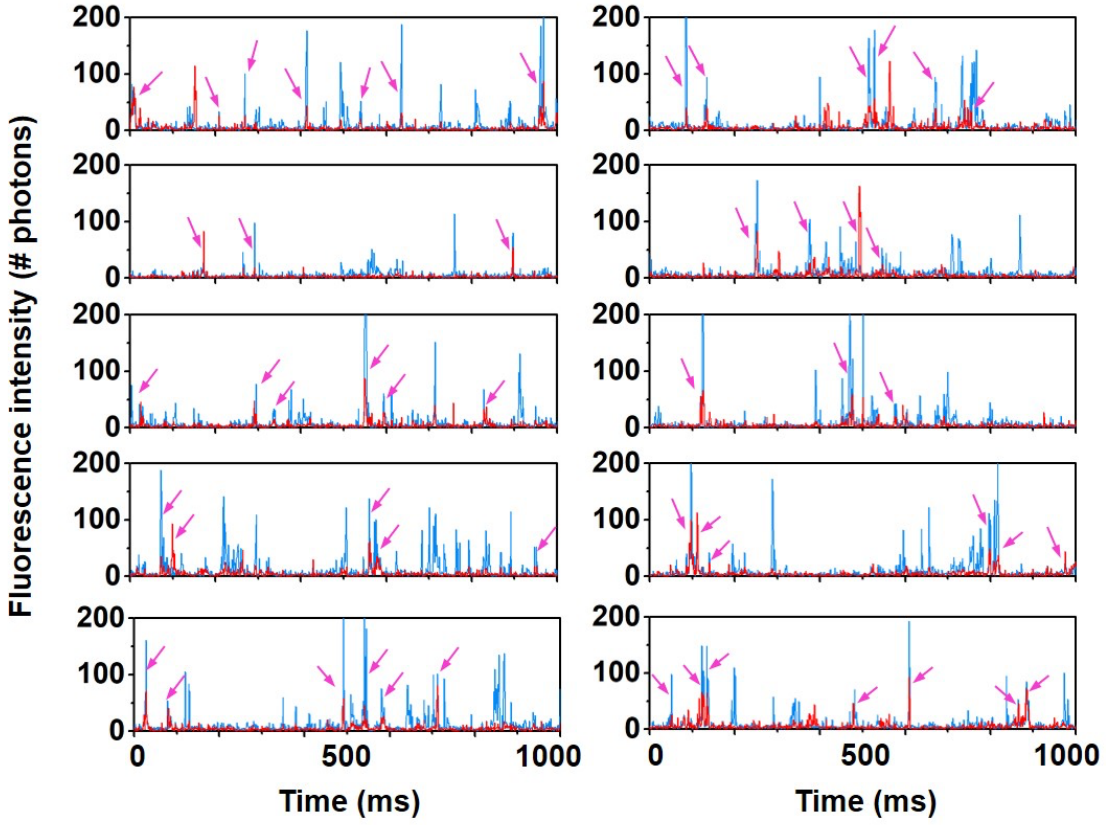

Supplementary Figure 14. **Single-particle conditions in time-resolved fluorescence spectroscopy experiments.** 10 representative 1-second intensity time traces (raw data) from a binding experiment with ~1 nM type B* oligomers (blue) and ~10 nM PSMα3 (red) are shown (note that concentrations are given as protein/peptide mass concentrations and that particle concentrations are significantly lower). Two-color coincident events that were intensity threshold-selected for fluorescence stoichiometry analysis are shown with pink arrows. The aggregate/complex-event frequency allows for optimal single-event selection and further burst-wise FRET and stoichiometry analysis, in full agreement with the aggregate mean volume occupancy (<*N*> far below 1) as explained elsewhere in the article.

Supplementary Table 1. **Computational analysis of redesigned variants.**

Name	Mutacion	Sequence	Activity	*H**	μ_H**	AGADIR	Net charge	Change in FoldX stability (kcal/mol)
**PSMα3**	-	MEFVAKLFKFFKDLLGKFLGNN	+++	0.54	0.56	2.65	2	-
**dPSMα3**	K9P_F11P	MEFVAKLFPFPKDLLGKFLGNN	-	0.57	0.44	0.40	1	3.67
**All_Leu**	Hydrophobic face to Leu	LELLAKLLKLLKDLLGKLLGNN	+++	0.57	0.58	66.14	2	-1.46
**All_Leu19**	Hydrophobic face to Leu without 3 C-ter residues	LELLAKLLKLLKDLLGKLL	+++	0.72	0.70	65.17	2	-1.46
**Scaffold_19**	Hydrophobic face to Leu without 3 C-ter residues A5E_G16K	LELLEKLLKLLKDLLKKLL	+++	0.62	0.77	77.68	2	-1.03
**Anionic scaffold**	Scaffold19 K6E_K12E	LELLEELLKLLEDLLKKLL	-	0.65	0.75	78.26	-2	0.65

* *H* indicates the mean hydrophobicity of the peptides.
** μ_H indicates the helical hydrophobic moment of the peptides.

Supplementary Table 2. **Identified human peptide candidates.** The screening of the human peptides database (EROP-Moscow) for cationic peptides with more than 10 residues, an AGADIR value > 2 and a helical hydrophobic moment (μ_H) > 0.2.

Peptide sequence	AGADIR	μ_H	Cysteines
>E02311|ANTIMICROBIAL PEPTIDE CATHELICIDIN LL37|HUMAN (HOMO SAPIENS), COMMOM CHIMPANZEE (PAN TROGLODYTES)<br><br>LLGDFFRKSKEKIGKEFKRIVQRIKDFLRNLVPRTES	5.10	0.521	No
>E02310|ANTIMICROBIAL PEPTIDE CATHELICIDIN FALL 39|HUMAN (HOMO SAPIENS), COMMOM CHIMPANZEE (PAN TROGLODYTES)<br><br>FALLGDFFRKSKEKIGKEFKRIVQRIKDFLRNLVPRTES	4.92	0.529	No
>E19967|SALUSIN BETA|HUMAN (HOMO SAPIENS)<br><br>AIFIFIRWLLKLGHHGRAPP	2.14	0.306	No
>E06260|ANAPHYLATOXIN C3A PEPTIDE LGE27|HUMAN (HOMO SAPIENS)<br><br>LGEACKKVFLDCCNYITKLRRQHARAS	5.63	0.493	Yes
>E06257|ANAPHYLATOXIN C3A PEPTIDE SLG25|HUMAN (HOMO SAPIENS)<br><br>SLGEACKKVFLDCCNYITELRRQHA	4.72	0.48	Yes
>E01232|MELANIN CONCENTRATING HORMONE|RAT (RATTUS NORVEGICUS), HUMAN (HOMO SAPIENS), MOUSE (MUS MUSCULUS)<br><br>DFDMLRCMLGRVYRPCWQV	3.82	0.406	Yes
>E06261|ANAPHYLATOXIN C3A PEPTIDE CNY21|HUMAN (HOMO SAPIENS)<br><br>CNYITELRRQHARASHLGLAR	4.17	0.241	Yes
>E05394|BETA DEFENSIN 4|HUMAN (HOMO SAPIENS)<br><br>EFELDRICGYGTARCRKKCRSQEYRIGRCPNTYACCLRKWDESLLNRTKP	3.09	0.32	Yes
>E04240|BETA DEFENSIN 6, HBD6|HUMAN (HOMO SAPIENS)<br><br>FFDEKCNKLKGTCKNNCGKNEELIALCQKSLKCCRTIQPCGSIID	3.23	0.231	Yes

References

1 Chen, S. W. et al. Structural characterization of toxic oligomers that are kinetically trapped during alpha-synuclein fibril formation. *Proc Natl Acad Sci* U S A **112**, E1994-2003 (2015).

2 Fusco, G. et al. Structural basis of membrane disruption and cellular toxicity by alpha-synuclein oligomers. *Science* **358**, 1440-1443 (2017).
