# Supplementary material for: α-Helical peptidic scaffolds to target α-synuclein toxic species with nanomolar affinity
Source: Nat Commun. 2021 Jun 18;12:3752. doi: 10.1038/s41467-021-24039-2 (PMC8213730; doi:10.1038/s41467-021-24039-2)
Supplement: Supplementary file 3 — Reporting Summary [file 41467_2021_24039_MOESM3_ESM.pdf]

nature research

Corresponding author(s): Nunilo Cremades, Irantzu Pallarès and Salvador Ventura

Last updated by author(s): May 17, 2021

Reporting Summary

Nature Research wishes to improve the reproducibility of the work that we publish. This form provides structure for consistency and transparency in reporting. For further information on Nature Research policies, see our Editorial Policies and the Editorial Policy Checklist.

Statistics

For all statistical analyses, confirm that the following items are present in the figure legend, table legend, main text, or Methods section.

n/a	Confirmed	
☐	☒	The exact sample size (*n*) for each experimental group/condition, given as a discrete number and unit of measurement
☐	☒	A statement on whether measurements were taken from distinct samples or whether the same sample was measured repeatedly
☐	☒	The statistical test(s) used AND whether they are one- or two-sided<br>*Only common tests should be described solely by name; describe more complex techniques in the Methods section.*
☒	☐	A description of all covariates tested
☐	☒	A description of any assumptions or corrections, such as tests of normality and adjustment for multiple comparisons
☐	☒	A full description of the statistical parameters including central tendency (e.g. means) or other basic estimates (e.g. regression coefficient) AND variation (e.g. standard deviation) or associated estimates of uncertainty (e.g. confidence intervals)
☐	☒	For null hypothesis testing, the test statistic (e.g. *F*, *t*, *r*) with confidence intervals, effect sizes, degrees of freedom and *P* value noted<br>*Give P values as exact values whenever suitable.*
☒	☐	For Bayesian analysis, information on the choice of priors and Markov chain Monte Carlo settings
☒	☐	For hierarchical and complex designs, identification of the appropriate level for tests and full reporting of outcomes
☒	☐	Estimates of effect sizes (e.g. Cohen's *d*, Pearson's *r*), indicating how they were calculated

Our web collection on statistics for biologists contains articles on many of the points above.

Software and code

Policy information about availability of computer code

Data collection

Time resolved fluorescence spectroscopy: Instrument- MT200 (PicoQuant) Software-SymphoTime64 (PicoQuant) (version 2.3).
Aggregation kinetics: Instrument- Victor3.0 Multilabel Reader (PerkinElmer) Software- PerkinElmer 2030.
Transmission electron microscopy: Instrument- TEM JEM-1400 (JEOL) Software- Gatan Digital Micrograph 1.8.
Circular dichroism: Instrument- Jasco J-815 CD spectrometer (Halifax) Software- Jasco spectra manager v2.
Confocal microscopy: Instrument- Leica TCS SP5 (Leica Microsystems) Software- Leica LAS AF (version 2.7.3.9723).
Atomic Force microscopy: Instrument- Bruker Multimode 8 (Bruker) Software- Gwyddion (version 2.48).
Dynamic light scattering: Intrument DynaPro NanoStar Software-Dynamics Software (version 6.12.03).
Fourier-Transform infrared spectroscopy: Instrument VERTEX 70 FTIR Spectrometer Software-OPUS (Bruker) (version 6.5), RAMOPN (NRC, National Research Council of Canada) (non version applicable) and Spectra-Calc-Arithmetic© (Galactic Inc.) (version A2.21).
Fluorescence spectroscopy: Instrument-Cary Eclipse Fluorescence Spectrophotometer (Varian).

Data analysis

Image J (1.52p), Graphpad Prism 7, OriginPro 9.1, SymphoTime64.

For manuscripts utilizing custom algorithms or software that are central to the research but not yet described in published literature, software must be made available to editors and reviewers. We strongly encourage code deposition in a community repository (e.g. GitHub). See the Nature Research guidelines for submitting code & software for further information.

Data

Policy information about availability of data

All manuscripts must include a data availability statement. This statement should provide the following information, where applicable:

- Accession codes, unique identifiers, or web links for publicly available datasets
- A list of figures that have associated raw data
- A description of any restrictions on data availability

The authors declare that all the data presented in this study are available in the paper or in the supplementary information.
Further raw data (i.e. Time traces of the time-resolved fluorescence spectroscopy) supporting the findings of this study are available from the corresponding author upon reasonable request.

Field-specific reporting

Please select the one below that is the best fit for your research. If you are not sure, read the appropriate sections before making your selection.

☒ Life sciences ☐ Behavioural & social sciences ☐ Ecological, evolutionary & environmental sciences

For a reference copy of the document with all sections, see nature.com/documents/nr-reporting-summary-flat.pdf

Life sciences study design

All studies must disclose on these points even when the disclosure is negative.

Sample size	No statistical methods were used to predetermine sample sizes. Sample sizes were determined based on previous publications on similar experiments. For time resolved fluorescence spectroscopy experiments (Seo et. al. Nat Commun, 2014, doi:10.1038/ncomms4724; (Tnimov et. al., JBC, 2012, doi:10.1074/jbc.M112.371294). For aggregation kinetics analysis (Pujols et. al. Proc Natl Acad Sci U S A, 2018, doi: 10.1073/pnas.1804198115), and for Cell-based assays (Cascella et. al. ACS Chem. Biol, 2019, doi: 10.1021/acschembio.9b00312).
Data exclusions	No data was excluded in the analysis.
Replication	All the experiments were repeated at least twice or thrice, if not stated otherwise in the methods section.<br>Due to the sensibility of time resolved fluorescence spectroscopy, repeated conditions are not exact replicates (small fluctuations in protein/aggregates concentration). All attempts of replication were successful and gave similar results.
Randomization	In cellular assays, imaged cells were selected randomly. In vitro assays did not require randomization since none of the recorded parameters are influenced by the observer.
Blinding	Blinding is not possible since the investigators who analyzed the data also performed the experiments.

Reporting for specific materials, systems and methods

We require information from authors about some types of materials, experimental systems and methods used in many studies. Here, indicate whether each material, system or method listed is relevant to your study. If you are not sure if a list item applies to your research, read the appropriate section before selecting a response.

Materials & experimental systems

n/a	Involved in the study	
☐	☒	Antibodies
☐	☒	Eukaryotic cell lines
☒	☐	Palaeontology and archaeology
☒	☐	Animals and other organisms
☒	☐	Human research participants
☒	☐	Clinical data
☒	☐	Dual use research of concern

Methods

n/a	Involved in the study	
☒	☐	ChIP-seq
☒	☐	Flow cytometry
☒	☐	MRI-based neuroimaging

Antibodies

Antibodies used	Antibody list (suppliers, catalogue numbers and dilutions):<br>ICC/IF<br>Anti-alpha-synuclein antibody. Abcam PLC, cat. ab155038, 1:200 dilution<br>Secondary:<br>Goat Anti-Rabbit IgG H&L (Alexa Fluor® 488). Abcam PLC, cat. ab150077, 1:1000 dilution

Validation	All the antibodies used in this study were commercial antibodies and were only used for applications, with validation procedures described on the following sites of the manufacturers:<br>Anti-alpha-synuclein antibody. Abcam PLC, cat. ab155038, 1:200 dilution<br>Rabbit polyclonal IgG 1:1000. https://www.abcam.com/alpha-synuclein-antibody-ab155038.html<br>Goat Anti-Rabbit IgG H&L (Alexa Fluor® 488). Abcam PLC, cat. ab150077, 1:1000 dilution. https://www.abcam.com/goat-rabbit-igg-hl-alexa-fluor-488-ab150077.html?productWallTab=Abreviews&applications=74%7C83

Eukaryotic cell lines

Policy information about cell lines

Cell line source(s)	SH-SY5Y (ATCC CRL-2266) human neuroblastoma were acquired from A.T.C.C. (VA, USA).
Authentication	Cell line was not authenticated by us.
Mycoplasma contamination	Cell lines tested negative for mycoplasma contamination.
Commonly misidentified lines (See ICLAC register)	No commonly misidentified lines were used in this study.
